# Shear Bond Strengths and Morphological Evaluation of Filled and Unfilled Adhesive Interfaces to Enamel and Dentine

**DOI:** 10.1155/2012/858459

**Published:** 2012-11-06

**Authors:** Vajihesadat Mortazavi, Mohammadhosein Fathi, Ebrahim Ataei, Niloufar Khodaeian, Navid Askari

**Affiliations:** ^1^Torabinejad Dental Research Center, Department of Operative Dentistry, School of Dentistry, Isfahan University of Medical Sciences, Isfahan 8174673461, Iran; ^2^Biomaterials Research Group, Department of Materials Engineering, Isfahan University of Technology, Isfahan 8415683111, Iran; ^3^Department of Operative Dentistry, School of Dentistry, Shahid Sadoughi University of Medical Sciences, Yazd 89195165, Iran; ^4^Dental Material Research Center, Department of Prosthodontics, School of Dentistry, Isfahan University of Medical Sciences, Isfahan 8174673461, Iran; ^5^Department of Prosthodontics, School of Dentistry, Shahid Sadoughi University of Medical Sciences, Yazd 89195165, Iran

## Abstract

In this laboratory study shear bond strengths of three filled and one unfilled adhesive systems to enamel and dentine were compared. Forty-eight extracted intact noncarious human mandibular molars were randomly assigned to two groups of 24 one for bonding to enamel and the other for bonding to dentine. Buccal and lingual surfaces of each tooth were randomly assigned for application of each one of filled (Prime & Bond NT (PBNT), Optibond Solo Plus (OBSP), and Clearfil SE Bond (CSEB)) and unfilled (Single Bond (SB)) adhesive systems (*n* = 12). A universal resin composite was placed into the translucent plastic cylinders (3 mm in diameter and 2 mm in length) and seated against the enamel and dentine surfaces and polymerized for 40 seconds. Shear bond strength was determined using a universal testing machine, and the results were statistically analyzed using two-way ANOVA, one-way ANOVA, *t*-test, and Tukey HSD post hoc test with a 5% level of significance.There were no statistically significant differences in bond strength between the adhesive systems in enamel, but CSEB and SB exhibited significantly higher and lower bond strength to dentine, respectively, than the other tested adhesive systems while there were no statistically significant differences between PBNT and OBSP.

## 1. Introduction

Dentine bonding systems continue to be developed at a rapid rate [1]. Satisfactory bonding to enamel can be achieved using the acid-etching technique [2], but dentine bonding is more difficult to achieve due to the wet tubular structure, permeability properties, and organic composition of dentinal substrate [3]. Recently there has been increasing interest in the incorporation of fillers into dentine adhesive systems, but the importance of filler particles is somewhat controversial [4, 5]. These fillers may include from conventional glass or silica fillers to nanometer-sized aerosil silica [6]. Recently, some researchers have incorporated nanoclay filler particles and hydroxyapatite nanorod fillers in dental adhesives to improve their properties [7–9]. Fillers have been added to some adhesive systems to improve bond strength by reinforcing the hybrid zone and reduce polymerizing shrinkage [10, 11]. However, increased filler loading increases viscosity of bonding system and may reduce its flow. If the addition of fillers prevents the adhesive from adapting optimally to the etched enamel and dentine surface and exposed collagen fibers, a suitable hybrid layer may not form [12], compromising bond strength and marginal integrity [13]. Inclusion of fillers in dentine adhesives increases their viscosity that tends to prevent overthinning of unfilled adhesive layers, thereby preventing incomplete polymerization caused by oxygen inhibition [14]. They may also provide stress relief capacities against shrinkage stresses generated during polymerization of resin-based restorative materials, in a way that is similar to the use of resin composite liners and flowable composites [15]. This intermediate layer that acts as an “elastic buffer” must have adequate properties to withstand the stresses of the oral environment [16], so the perceived advantages of filled adhesives as stress buffers remain unpredictable [17]. The optimum filler level for maximum increase in bond strength may be affected by several factors. They will include the size, shape, content of filler particles, and the surface properties of the fillers (hydrophilic versus hydrophobic) [18]. The purpose of the current study was to compare shear bond strengths of three filled and one unfilled adhesive systems to enamel and dentine. The null hypothesis was that shear bond strength of filled and unfilled adhesive systems to enamel and dentine was not different. 

## 2. Materials and Methods 

Forty-eight extracted noncarious human mandibular molars, which had been stored for less than four weeks in 0.2% thymol, were selected and cleaned. The teeth were randomly assigned to two enamel and dentine groups, with 24 teeth in each group. In dentine group, superficial dentine was exposed by removing the buccal and lingual enamel using diamond bur (852.FG.010, Jota, Switzerland) under running water as coolant. Then the teeth were mounted in self-curing acrylic resin (Flash Acrylic, Yates Motloid, Chicago, IL, USA) to a level 1 mm below the CEJ of every tooth. Buccal and lingual surfaces of teeth in enamel and dentine groups were randomly selected for application of each bonding system used in this study. Before application of dentine bonding systems, enamel and dentine surfaces were polished by 600 grit silicone paper under running water to create standard smear layer on each tooth surface. After the preparation of tooth surfaces, adhesive systems were applied to surfaces according to their manufacturer's instructions. The adhesive systems and the resin composite used in the present study and their compositions are listed in Table 1. Translucent plastic cylinders (3 mm in diameter and 2 mm in length) were filled with Filtek Z100 light cure resin composite (3 M ESPE dental products, St. Paul, MN, USA) and bonded to enamel and dentine surfaces and irradiated with blue phase LED curing light (Ivoclar Vivadent, Schaan, Liechtenstein) for 40 seconds. The specimens were then stored in deionized water at 37°C and plastic cylinders removed after an hour using a feather blade. Twenty four hours after bonding, shear bond strengths were determined with a universal testing machine (Dartec, Series TLCLO, England) using a knife-edged loading head just contacting the interface of the enamel/dentine and resin composite column at a cross-head speed of 1 mm/min. Bond strength values were obtained from the specimens in each group. All procedures were carried out by one operator. Two-way ANOVA was used to statistically analyze the differences in shear bond strength values between all the test groups. One-way ANOVA along with Tukey HSD post hoc test was used to statistically analyze the differences between bond strength values of different adhesive systems in either enamel or dentine groups. *t*-test was used for pair-wise comparisons when indicated. A 5% level of significance was adopted. 

### 2.1. Interface Micromorphology

Another 8 specimens, 4 for dentine and 4 for enamel, were used for scanning electron microscopy (SEM) examination of resin enamel/dentine interfaces. After preparation of buccal or lingual surface of each tooth in the same manner as the bonding procedure for adhesive systems, Filtek Z100 resin composite was placed in buccal or lingual tooth surface in 1 mm thickness and cured for 40 seconds. Then teeth were sectioned perpendicular to the bonded interface (buccolingually) using a low-speed Isomet saw (Buehler diamond wafering blade 15 HC, Buehler, USA) under running water as coolant obtaining 16 interface sections. Each interface was finished with a 1000 grit silicon carbide paper under water and polished with 6, 3, 1, and 0.25 *μ*m diamond paste using a polish cloth under water. The interface sections were rinsed between the polishing steps with water and debris and paste removed ultrasonically for 5 min. For inspection of resin tags in dentine interfaces, dentine samples were etched with 6 N/HCl for 30 seconds and then rinsed. Samples were immersed in 2.5% NaHClO for 10 minutes to remove collagen fibers and other organic parts of dentine. After rinsing, all samples were placed in dry environment for 24 hours, and then each section was sputter coated with gold (BAL-TEC, Sputter coater, Netherlands) and observed by SEM (XL 30, Philips, Netherlands).

## 3. Results

Shear bond strength value of each specimen and the mean and standard deviation value of each group are shown in Tables 2 and 3, respectively. Two-way ANOVA revealed significant differences of shear bond strength values between enamel and dentine groups (*P* < 0.001). Maximum and minimum shear bond strength values in enamel groups were found in Prime & Bond NT (PBNT) (22.74 ± 4.45 MPa) and Clearfil SE Bond (CSEB) (18.84 ± 4.31 MPa) groups, respectively. One-way ANOVA revealed no significant differences in shear bond strength values between adhesive systems in enamel (*P* = 0.127), but shear bond strength values were significantly different in dentine group (*P* < 0.001). Maximum and minimum of shear bond strength values in dentine group were observed in CSEB (18.19 ± 4.43 MPa) and Single bond (SB) (9.53 ± 2.02 MPa) groups, respectively.

Tukey HSD post hoc test (Table 4) revealed no significant differences between shear bond strength values of PBNT and Optibond Solo Plus (OBSP), but there were significant differences between PBNT, OBSP, and CSEB with SB. Shear bond strength values of PBNT and OBSP were also significantly different with CSEB. *t*-test showed that there were significant differences between shear bond strength values of PBNT, OBSP, and SB for enamel and dentine (*P* < 0.001 for all 3 adhesives), but, for CSEB, shear bond strength values were not significantly different for enamel and dentine (*P* = 0.719).

**Figure 1:**
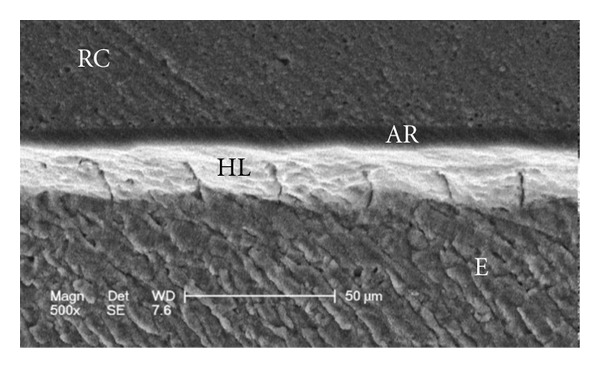
Scanning electron micrograph of the resin-enamel interface bonded with PBNT (500x). RC: resin composite; AR: adhesive resin; HL: hybrid layer; E: enamel.

**Figure 2:**
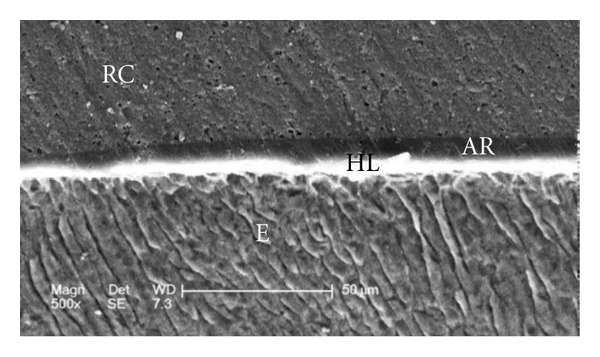
Scanning electron micrograph of the resin-enamel interface bonded with OBSP (500x). RC: resin composite; AR: adhesive resin; HL: hybrid layer; E: enamel.

**Figure 3:**
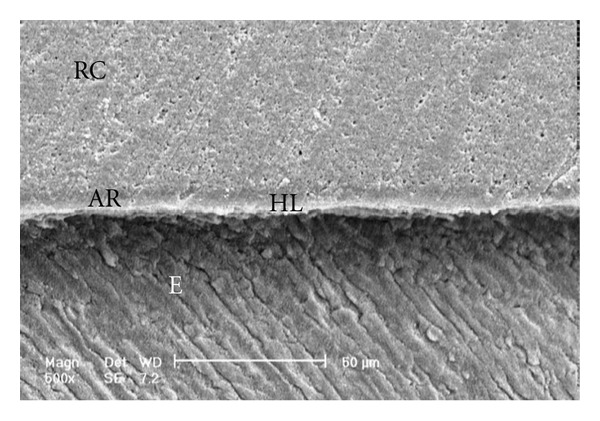
Scanning electron micrograph of the resin-enamel interface bonded with SB (500x). RC: resin composite; AR: adhesive resin; HL: hybrid layer; E: enamel.

**Figure 4:**
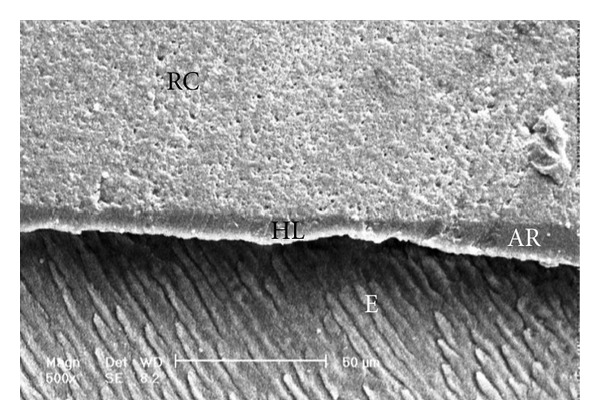
Scanning electron micrograph of the resin-enamel interface bonded with CSEB (500x). RC: resin composite; AR: adhesive resin; HL: hybrid layer; E: enamel.

Scanning electron micrographs of enamel/adhesive or dentine/adhesive interfaces are shown in Figure 1 to Figure 11. For all adhesive systems tested in this study, the enamel/adhesive interfaces showed good adaptation and gaps/artifacts were not found in theme (Figures 1, 2, 3, and 4). The two filled etch-and-rinse adhesive systems, OBSP and PBNT, showed relatively thicker adhesive resin layer (AR) and hybrid layer (HL) compared to the unfilled etch-and-rinse adhesive system SB and the filled self-etch adhesive system, CSEB. 

**Figure 5:**
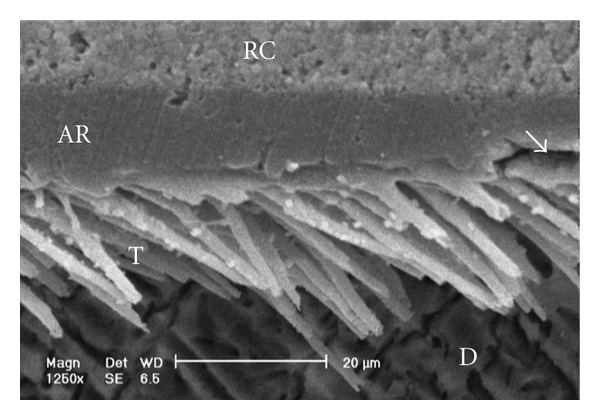
Scanning electron micrograph of the resin-dentin interface bonded with PBNT (1250x). Numerous resin tags and a small gap (arrow) within the adhesive resin layer are visible. RC: resin composite; AR: adhesive resin; HL: hybrid layer; D: dentin; T: tag.

**Figure 6:**
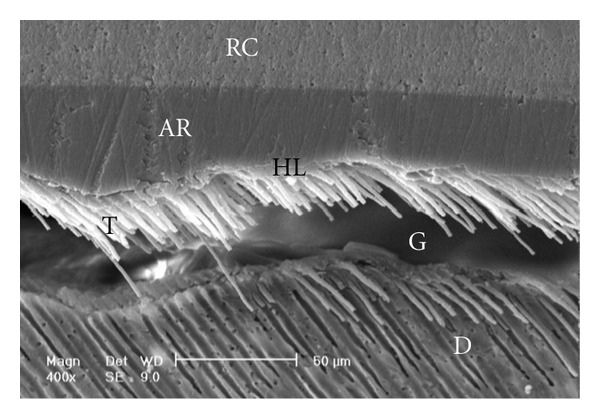
Scanning electron micrograph of the resin-dentin interface bonded with PBNT (400x). An adhesive defect (Gap; G) can be seen in this image. Several reasons may contribute to this event and polymerization deficiency may be one of them. Numerous resin tags have been detached from dentinal tubules. RC: resin composite; AR: adhesive resin; HL: hybrid layer; D: dentin; T: tag.

**Figure 7:**
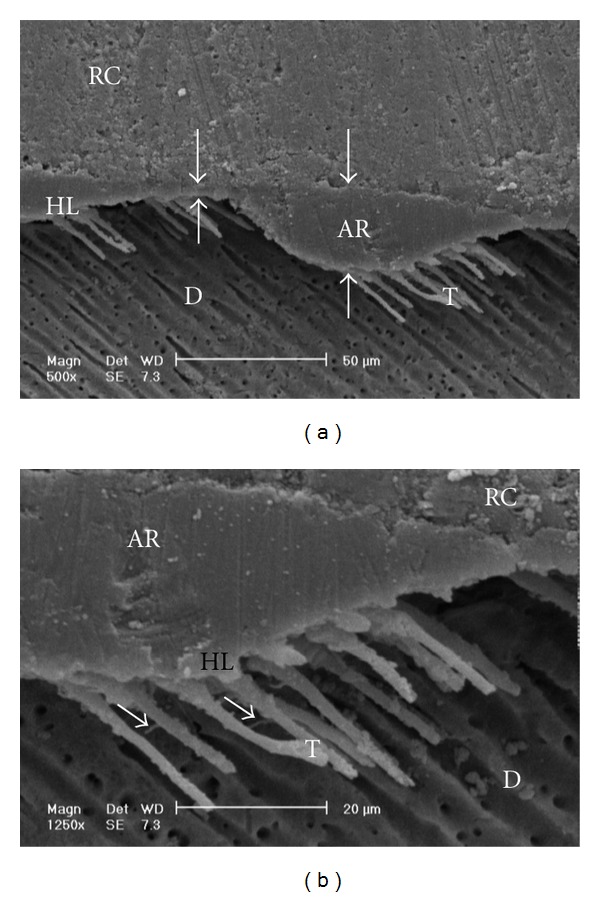
(a) Scanning electron micrograph of the resin-dentin interface bonded with OBSP (500x). Resin tags of the adhesive resin layer are visible. The adhesive layer thickness is not uniform in this image (opposing arrows). RC: resin composite; AR: adhesive resin; HL: hybrid layer; D: dentin; T: tag. (b) Scanning electron micrograph of the resin-dentin interface bonded with OBSP (1250x). Resin tags and lateral branches (arrows) of the adhesive resin layer are visible. RC: resin composite; AR: adhesive resin; HL: hybrid layer; D: dentin; T: tag.

**Figure 8:**
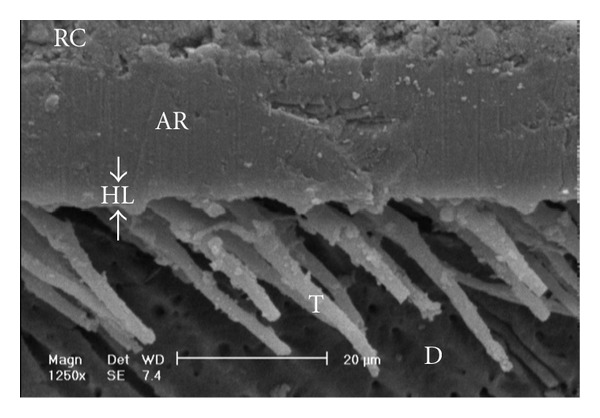
Scanning electron micrograph of the resin-dentin interface bonded with OBSP (1250x). Numerous resin tags of the adhesive resin layer are visible. A thin hybrid layer could be seen in this image (between arrowheads). RC: resin composite; AR: adhesive resin; HL: hybrid layer; D: dentin; T: tag.

**Figure 9:**
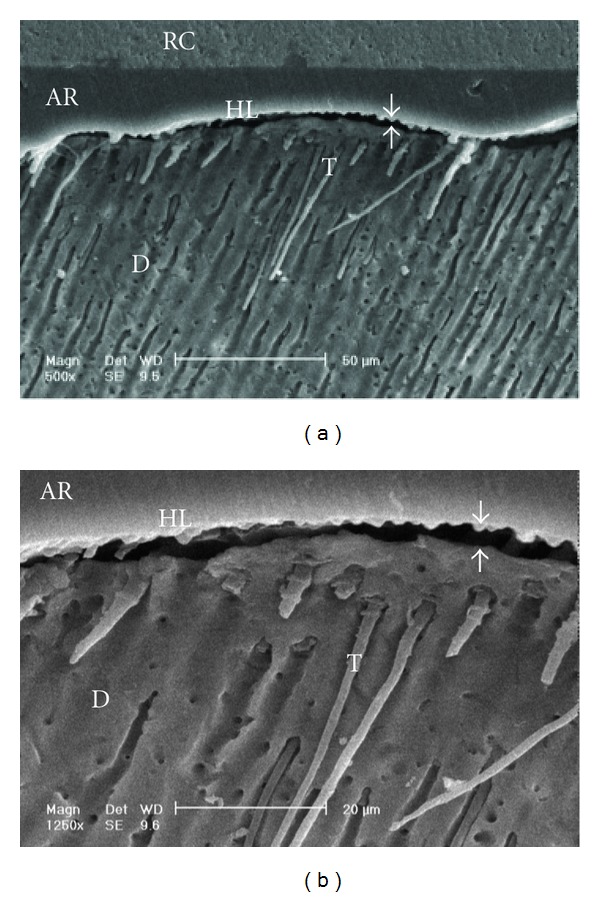
(a) Scanning electron micrograph of the resin-dentin interface bonded with SB (500x). A few long and short resin tags are visible, and some of tags are seen in dentinal tubules. Continuous gap could be seen in this image (opposing arrows). RC: resin composite; AR: adhesive resin; HL: hybrid layer; D: dentin; T: tag. (b) Scanning electron micrograph of the resin-dentin interface bonded with SB (1250x). Note to the continuous gap beneath the hybrid layer (opposing arrows). RC: resin composite; AR: adhesive resin; HL: hybrid layer; D: dentin; T: tag.

**Figure 10:**
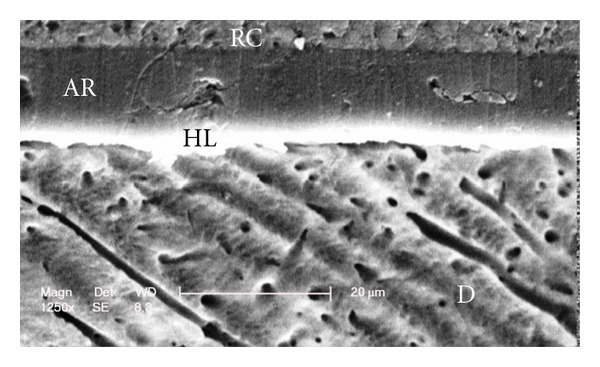
Scanning electron micrograph of the resin-dentin interface bonded with CSEB (1250x). Uniform adhesive and hybrid layer formation and good adaptation of resin-dentin interface could be seen. No resin tag formation is visible. RC: resin composite; AR: adhesive resin; HL: hybrid layer; D: dentin.

**Figure 11:**
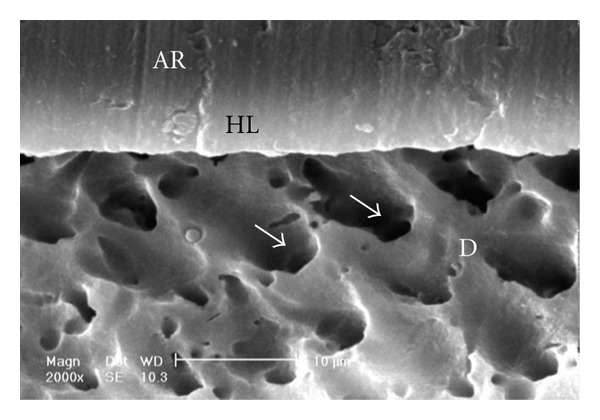
Scanning electron micrograph of the resin-dentin interface bonded with CSEB (2000x). Good adaptation of resin-dentin interface and orifices of the dentinal tubules (arrows) could be seen. No resin tag formation is visible. AR: adhesive resin; HL: hybrid layer; D: dentin.

Gaps/artifacts were observed in dentinal areas of resin/dentine interfaces of etch-and-rinse adhesive systems (Figures 5, 6, 7, 8, and 9), but CSEB self-etch adhesive samples did not reveal any gaps at the resin/dentine interface (Figures 10 and 11)

## 4. Discussion

It is difficult to evaluate the effect of fillers in dentine adhesives with dissimilar resin composition [19]. Filled adhesives were expected to act as an intermediate shock-absorbing elastic layer between resin composite and tooth surface, thus increasing the bond strength [5]. Several studies evaluated comparisons between commercially available filled and unfilled adhesives. However, the advantages of these adhesives as stress buffers remain unpredictable [17, 20]. Filler type, size, shape, surface characteristics, and interaction with the resin matrix and various solvents in adhesives may affect the bond strength [18]. A number of studies have investigated the bonding ability of adhesive systems to either enamel, dentine, or both. Most clinically prepared cavities are complex in design and include not only areas of exposed enamel and superficial dentine, but also deep dentinal areas. Since many different adhesive systems are on the market today, it is desirable to use adhesive systems that produce high uniform bond strengths to all of these dental hard tissues. In the present study three commercial etch-and-rinse adhesive systems (PBNT, OBSP and SB) and one self-etch adhesive system (CSEB) were evaluated. Among these adhesive systems only SB was unfilled.

### 4.1. Enamel Bond Strength

Enamel adhesion by means of phosphoric acid etching has become an accepted technique in restorative dentistry [21]. While traditionally 30–40% phosphoric acids have generally been used in etch-and-rinse adhesive systems, self-etching adhesives are composed of acidic monomers rather than phosphoric acid [22]. The mild aggressiveness of these acidic monomers could result in minor modifications and less enamel loss, which, in turn, could affect resin adaptation [23]. Therefore enamel bond strength of etch-and-rinse adhesive systems is expected to be superior to that of self-etching adhesives [24, 25]. The higher bond strengths for acid-etched enamel can be explained by the more microretentive enamel surface obtained when enamel is etched with phosphoric acid as compared to when enamel is etched by the self-etch adhesives. Several authors have reported that mild self-etch adhesives demineralized enamel shallowly, resulting in a very thin microretentive pattern without formation of distinct macro- and microresin tags [26]. While self-etching adhesives show shallow etching patterns, in several studies, their bond strengths to enamel were found to be similar to etch-and-rinse adhesive systems [27, 28]. One another study reported that only CSEB, which includes 10-MDP(10-methacryloxydecyl dihydrogen phosphate) functional monomers in its composition, achieves high enamel bond strength, which was similar to the etch-and-rinse systems [29]. The self-etch adhesive in this study belongs to the category of mild self-etch adhesives with pH of approximately 2. Although in the present study CSEB produced lower bond strengths than three other etch-and-rinse adhesives in enamel, but the differences were not significant. Additionally, SB which does not have fillers in its composition revealed nearly the same bond strength in comparison to other filled etch-and-rinse systems and superior to the filled self-etch adhesive system tested in this study, so it seems that presence of filler in adhesive systems tested did not significantly affect the bond strengths of adhesives to enamel. On the other hand, the formation of micro- and macroretentive characteristics in enamel with phosphoric acid etching and/or chemical reaction to hydroxyapatite with functional monomers such as 10-MDP seem to be more important factors for bonding to enamel than presence or absence of fillers in adhesive compositions.

### 4.2. Dentine Bond Strength

Dentine is known to be a less-favorable substrate than enamel for resin bonding due to its high organic content and the presence of fluid and the odontoblastic process in dentine tubules [30, 31]. For adhesive materials with aggressive etching effects, the dentine collagen network would be deprived of the hydroxyapatite coating. This would mean the absence of an effective chemical interaction and hence inadequate hybridization with dentine. Consequently, the bond strength to dentine would undergo a significant loss especially after storage for a long time due to hydrolysis of collagen fibrils [32]. However, two-step self-etching materials such as CSEB are unlike bonding systems that have a separate, aggressive acid-etching step. CSEB is received and perceived as one of the most reliable adhesive systems and has been chosen as the reference bonding system in numerous studies [33–35]. With CSEB which is a two-step self-etching system, etching and penetration of the primer monomers occur simultaneously [36]. Some researchers have highly lauded such two-step self-etching systems for their simultaneous monomer penetration and complete impregnation of the collagen network which leads to the formation of a homogenous and void-free interfacial zone that improves the quality of the hybrid layer and contributes to long-term sealing of the dentine surface [37, 38]. Long-term clinical evaluation of CSEB suggested that more aggressive etching was not essential for the overall clinical performance of the restorations [39]. In fact, mild acid etching enables the bonding substrate to maintain a higher mineral content for chemical interactions [40]. In addition, mild acid etching of dentine has the advantage of the occlusion of the dentinal tubules and consecutively decreasing dentine permeability and fluid movement, which may otherwise lead to hydrolytic degradation and failure of the bond [41].

According to the manufacturer, CSEB included a hydrophilic acidic monomer, 10-MDP, as the functional monomers. Susceptibility of resin components to hydrolysis has been identified as a cause for decreased bond strength. It has been suggested that outstanding hydrolytic stability of MDP and its additional chemical interaction with the enamel and dentine contributed to superior bonding to enamel and dentine. MDP has a special molecular structure that enables chemical interaction with residual hydroxyapatite after etching, and the produced chemical salt also exhibits hydrolytic stability [42]. In the current study it was found that, for dentine, CSEB had significantly higher bond strengths than other adhesive systems tested. Its good performance on dentine can be explained by its specific and adapted composition and the use of the functional monomer 10-MDP, which has been shown to exhibit highly chemical interaction capacity to hydroxyapatite [43]. Significantly lower bond strengths of etch-and-rinse adhesive systems to dentine may be attributed to suboptimally infiltration of the demineralized collagen network and subsequent poor adaptation of the bonding resin to the collagen fibrils [44]. Among the remaining three etch-and-rinse adhesive systems tested in the present study, SB which does not have filler in its composition showed significantly lower bond strength to dentine. The presence of fillers may produce a sufficiently thick resin film that stabilizes the hybrid layer and provide an elastic buffer zone that compensates for shrinkage stress during polymerization [45]. Miyazaki et al. [46] reported that a 10% filler content in adhesives was necessary to increase bond strength. For dentine, filled adhesives used in this study (CSEB, OBSP, and PBNT) revealed significantly higher bond strength than SB which was an unfilled adhesive system, but the differences between the bond strengths of PBNT and OBSP were not significant. It seems that, for bonding to dentine, filled adhesives are more effective than unfilled adhesives and also two-step self-etching adhesives perform more effectively than etch-and-rinse adhesive systems. Bond durability of CSEB is ensured by the presence of MDP functional monomers and filler particles and formation of relatively thicker layer that serve as an elastic buffer zone during polymerization of resin composite [47]. Finally, since the adhesives used in this study contained different solvents, it is possible that the solvent (water, ethanol or acetone) produces a significant effect on the viscosity of the adhesive which affects its ability to adapt to the dentine surface effectively, which in turn could influence bond strength. If it was possible to use adhesive systems with similar compositions and different filler contents, the results could have been interpreted more reliably. 

### 4.3. Scanning Electron Microscopy (SEM) Analysis

As the hybrid layer is visualized under SEM, which is only possible by sectioning the resin dentine/enamel interfaces, it is possible that even a slight inclination of the cutting direction makes the hybrid layer appear thicker [48].

Gaps/artifacts were observed in dentinal areas of adhesive/dentine interfaces, indicating that dentine bonding is likely to be influenced by more factors than enamel [49]. The observed gap in these areas may have originated from or may have been increased by air drying and desiccating the specimens for SEM observation. However, since such gaps or cracks were not evident only in self-etch adhesive system CSEB, and since all specimens were treated in the same manner, they may have been attributed to poorly polymerized hybrid/adhesive layers.

Numerous resin tags (T) and lateral tags indicated that the smear layer was sufficiently dissolved by the phosphoric acid etching of etch-and-rinse adhesive systems [50]. Resin tags were not seen in scanning electron micrographs of CSEB sections which may be due to parallel path of section to the dentinal tubules.

## 5. Conclusions

According to the results and limitations of the present study, it can be concluded the following.

1. Etch-and-rinse dentine bonding systems produce reliable bonding to enamel whether they include fillers in their composition or not.
2. Two-step self-etch adhesives are more effective than etch-and-rinse systems in bonding to dentine.
3. Filled dentine bonding agents produce more reliable bonding to dentine than unfilled adhesive systems.

## Figures and Tables

**Table 1:** Materials used in the present study and their composition.

Material	Composition	Manufacturer
Prime & bond NT (PBNT)	PENTA, UDMA resin, resin R5-62-1, T-resin, D-resin, nanofiller, initiators, stabilizer, Cetylamine hydrofluoride, acetone.	Dentsply/De Trey GmbH, Konstanz, Germany LOT no. 0611601781
Optibond Solo Plus (OBSP)	Bis-GMA, GPDM, HEMA, silica, barium glass, sodium hexafluorosilicate, ethanol, water.	Kerr Corp, Orange, CA, USA LOT no. 07-1057
Single bond (SB)	HEMA, Bis-GMA, dimethacrylates, ethanol, water, polyalkenoic acid, copolymer, initiator.	3M Dental Products, St. Paul, MN, USA LOT no. 2006 0299
Clearfil SE bond (CSEB)	Primer: 10-methacryloyloxydecyl dihydrogen phosphate, 2-hydroxyethyl methacrylate, hydrophilic dimethacrylate, di-camphorquinone, N,N-diethanol-p-toudine, water. Bond: 10-methacryloyloxydecyldihydrogen. Phosphate, N,N-diethanol-p-toludine, 2-hydroxyethylmethacrylate, Bis-phenol A diglycidylmethacrylate, silanated colloidal silica, hydrophobic dimethacrylate, dicamphorquinone.	Kuraray Co, Osaka, Japan LOT no. 51435
Filtek Z100 composite	Bis-GMA, TEGDMA, zirconium/silica filler.	3M Dental Products, St Paul, MN, USA LOT no. 20070829

PENTA: pentaacrylate ester; TEGDMA: triethylene glycol-dimethacrylate; Bis-GMA: bysphenyl methacrylate; UDMA: urethane dimethacrylate; HEMA: 2-hydroxyethyl methacrylate; GPDM: glycerophosphoric acid dimethacrylate.

**Table 2:** Shear bone strength (MPa) for different adhesive systems on enamel and dentine.

Adhesive system		Shear bond strength											
Prime & bond NT	Enamel	23.4	25.0	23.4	24.9	29.6	30.1	20.8	21.9	23.0	16.1	18.2	16.7
	Dentine	18.8	12.4	15.6	19.7	14.3	9.1	11.7	15.5	15.7	14.3	10.4	11.5
Opti bond solo plus	Enamel	19.3	20.8	18.2	15.6	23.4	18.2	27.5	18.7	25.5	26.0	23.4	28.6
	Dentine	13.0	15.6	17.1	12.0	11.5	18.3	12.9	16.6	10.4	10.9	10.4	17.7
Single bond	Enamel	16.1	18.2	23.4	22.9	13.8	20.8	18.2	28.6	25.2	20.8	23.3	23.5
	Dentine	8.3	9.3	11.2	10.3	11.7	13.1	9.3	10.0	5.6	9.9	7.8	7.8
Clearfil SE bond	Enamel	13.6	18.2	11.2	20.9	15.5	23.4	18.1	25.6	21.4	20.8	15.0	23.4
	Dentine	18.6	23.0	16.3	22.7	23.3	15.7	10.3	14.6	19.2	12.9	17.6	24.0

**Table 3:** Mean and standard deviation values for shear bonding strength of 4 adhesive systems tested to enamel and dentine (MPa).

Material	Enamel	Dentin
	mean ± SD	mean ± SD
Prime & bond NT	22.74 ± 4.45	14.08 ± 3.22
Optibond solo plus	22.09 ± 4.22	13.86 ± 3.00
Single bond	21.23 ± 4.12	9.53 ± 2.02
Clearfil SE bond	18.84 ± 4.31	18.19 ± 4.43

**Table 4:** Tukey HSD post hoc test results (*P* values) for differences in shear bond strength values between experimental groups in dentine.

Materials	Clearfil SE bond	Single bond	Optibond solo plus
Prime & bond NT	0.031*	0.003*	0.999
Optibond solo plus	0.021*	0.005*	
Single bond	<0.001*		

^∗^Significant difference.
